# Gender, Anaemia, and Azotaemia as Correlates of Thyroid Dysfunction in Chronic Kidney Disease: A Cross-Sectional Study From North India

**DOI:** 10.7759/cureus.106311

**Published:** 2026-04-02

**Authors:** Anirban Bhaumik, Amit Johari, Aninda Debnath, Santosh Upadhyay

**Affiliations:** 1 Internal Medicine, Dr. Baba Saheb Ambedkar Medical College and Hospital, New Delhi, IND; 2 Community Medicine, Maulana Azad Medical College, New Delhi, IND; 3 Internal Medicine, Maharaja Agrasen Hospital, New Delhi, IND

**Keywords:** anaemia, azotaemia, chronic kidney disease, hypothyroidism, subclinical hypothyroidism

## Abstract

Background and objective

Thyroid dysfunction is a well-recognised but under-investigated complication of chronic kidney disease (CKD). While the overall prevalence of hypothyroidism in CKD is documented, its association with specific clinical variables, particularly including gender, haemoglobin (Hb), and azotaemia, has not been systematically characterised in populations from the Indian subcontinent. Hence, this study was designed to identify the correlates of thyroid dysfunction in CKD patients on conservative management.

Methods

A cross-sectional observational study was conducted involving 100 CKD patients (age ≥ 18 years) attending the outpatient and inpatient wards of Dr. Baba Saheb Ambedkar Medical College and Hospital (Dr. BSAH), New Delhi, between 2019 and 2021. Patients on dialysis, those with nephrotic syndrome, pregnancy, liver disease, or medications known to alter thyroid function were excluded. CKD was staged using the Kidney Disease: Improving Global Outcomes (KDIGO) criteria and the four-variable Modification of Diet in Renal Disease (MDRD) formula. Serum free triiodothyronine (FT3), free thyroxine (FT4), and thyroid-stimulating hormone (TSH) were measured by an enzyme-linked immunosorbent assay (ELISA). Statistical analysis was performed using IBM SPSS Statistics version 20 (IBM Corp., Armonk, NY), employing the chi-square test, one-way analysis of variance (ANOVA), the independent samples t-test, the Pearson correlation, and binary odds ratio (OR) calculation.

Results

The mean age of the cohort was 51.32 ± 11.89 years; 72 (72%) were male and 28 (28%) female. CKD stage 5 was the most common stage (44 (44%)). Total hypothyroidism prevalence was 46% (subclinical hypothyroidism (SCH): 24 (24%), overt hypothyroidism (OH): 22 (22%), low T3 syndrome: nine (9%)). Female patients had a significantly higher hypothyroidism rate (20 (71.4%) vs 26 (36.1%); χ² = 10.335, OR = 4.42, 95% CI: 1.71-11.44, p = 0.003) and higher mean TSH (5.84 vs 4.41 μIU/ml; t = −2.116, p = 0.036). Overt hypothyroidism was associated with the lowest Hb (7.97 g/dl; ANOVA F = 6.349, p = 0.001) and the highest blood urea (152.03 mg/dl; ANOVA F = 3.24, p = 0.024). Hb correlated negatively with TSH (r = −0.236, p = 0.018) and positively with estimated glomerular filtration rate (eGFR) (r = 0.516, p < 0.001). Advanced CKD stage 5 was significantly associated with a higher risk of hypothyroidism (OR = 2.60, 95% CI: 1.15-5.85, p = 0.034). Comorbidities (type 2 diabetes mellitus (T2DM), hypertension (HTN)) and age were not significantly associated with thyroid dysfunction.

Conclusions

Female gender and advanced CKD stage show the strongest associations with hypothyroidism in this cohort. Overt hypothyroidism is associated with more severe anaemia and higher azotaemia, beyond what is explained by CKD stage alone. These findings support targeted thyroid function testing in CKD patients; prospective studies are warranted to establish the clinical benefit of such a strategy.

## Introduction

Chronic kidney disease (CKD) is a global health burden affecting an estimated 10-15% of the adult population worldwide and representing a leading cause of morbidity and end-stage renal disease [1]. In India, the prevalence of CKD is rising sharply, driven by the dual epidemics of type 2 diabetes mellitus (T2DM) and hypertension (HTN), with studies reporting a CKD prevalence of 17.2% in the general adult population [2]. As CKD progresses, it affects virtually every organ system, and non-renal complications, including endocrine dysfunction, significantly contribute to its overall morbidity and mortality.

Among endocrine complications, thyroid dysfunction deserves particular attention in CKD. The kidney plays a central role in peripheral thyroid hormone metabolism: it participates in the deiodination of thyroxine (T4) to triiodothyronine (T3), excretes iodine, and influences the hypothalamo-pituitary-thyroid (HPT) axis through uraemia-related dysregulation [3]. As a result, a spectrum of thyroid abnormalities, including subclinical hypothyroidism (SCH), overt hypothyroidism (OH), and the low T3 syndrome, is commonly encountered in CKD patients.

The reported prevalence of hypothyroidism in CKD varies widely in the literature, ranging from 20% to 60%, depending on the population studied, the stage of CKD, and the diagnostic criteria employed [4-6]. Pioneering studies by Ramirez et al. first described altered thyroid hormone metabolism in uraemic patients, demonstrating low T3 and elevated reverse T3 as hallmarks of CKD-associated thyroid dysfunction [7]. Subsequent work by Kaptein et al. documented the progressive decline in serum T3 with worsening renal function and confirmed partial TSH suppression in dialysis patients [8].

Despite this body of literature, several clinically important questions remain incompletely addressed. Firstly, the role of gender as a correlate of hypothyroidism in CKD is inconsistently reported, with some studies attributing the higher prevalence of hypothyroidism in females solely to pre-existing autoimmune thyroid disease, while others suggest a more complex interplay with uraemia [9,10]. Second, the relationship between the degree of anaemia, a universal feature of CKD, and thyroid hormone status has not been systematically quantified. Both anaemia and hypothyroidism independently impair cardiac output and oxygen delivery; their coexistence may produce a compounding haematological effect. Third, the association between azotaemia (elevated blood urea) and thyroid hormone levels, beyond what is explained by eGFR alone, remains poorly characterised in the Indian CKD population.

This study was designed with three principal objectives: (i) to determine the prevalence and pattern of thyroid dysfunction across CKD stages 3 to 5 in an Indian hospital-based cohort; (ii) to evaluate gender as an independent predictor of hypothyroidism; and (iii) to characterise the relationships between haemoglobin (Hb), blood urea, serum creatinine, and thyroid dysfunction indices, with the aim of identifying clinically actionable screening targets in this high-risk population.

## Materials and methods

Study design and setting

This was a cross-sectional observational study conducted in the Department of General Medicine at Dr. Baba Saheb Ambedkar Medical College and Hospital (Dr. BSAH), New Delhi, India, a 1200-bed tertiary care government teaching hospital. The study was conducted over approximately two years, from 2020 to 2021, and was approved by the Institutional Ethics Committee (IEC) (IEC meeting date: 08/11/2019; reference no. F 5(59)/2017/BSAH/DNB/Committees/32148). The IEC is duly registered with the Drug Controller General of India (DCGI). Written informed consent was obtained from all participants.

Sample size

The Department of General Medicine evaluates approximately 50-60 patients with CKD on conservative management each year, yielding an estimated finite eligible population of 120 patients across the two-year study period (2020 to 2021). Sample size was calculated using the finite population correction formula. Assuming an expected prevalence of hypothyroidism in CKD of 50%, an absolute precision of 5%, and α = 0.05, (Z = 1.96), the infinite population estimate was n₀ = Z²pq/d² = 384. Applying the finite population correction, n = n₀N/(n₀ + N − 1) = 384 × 120/503 ≈ 92. The minimum required sample size was therefore 92 patients. A total of 100 patients were enrolled through consecutive sampling over the study period.

Study population

A total of 100 patients fulfilling the inclusion criteria were enrolled consecutively from both the outpatient and inpatient departments. The inclusion criteria were as follows: (i) age ≥ 18 years; (ii) CKD diagnosed based on persistent structural or functional kidney abnormalities for more than three months, in accordance with the Kidney Disease: Improving Global Outcomes (KDIGO) 2012 guidelines; and (iii) currently on conservative (non-dialysis) management. Patients were excluded if they were on any form of renal replacement therapy (haemodialysis or peritoneal dialysis); had nephrotic syndrome; had a prior known diagnosis of hypothyroidism or were receiving thyroid hormone replacement; were pregnant or in the postpartum period; had liver cirrhosis or chronic liver disease; or were receiving medications known to alter thyroid function (amiodarone, lithium, glucocorticoids, radiocontrast agents, or anti-thyroid drugs).

CKD staging

CKD staging was performed using KDIGO 2012 criteria. Per KDIGO 2012, CKD is defined as persistent abnormalities of kidney structure or function for more than three months, evidenced by kidney damage markers (albuminuria, urine sediment abnormalities, electrolyte disorders due to tubular dysfunction, histological or structural abnormalities, or history of kidney transplantation) or a reduced estimated glomerular filtration rate (eGFR) below 60 ml/min/1.73m². The eGFR was calculated using the four-variable Modification of Diet in Renal Disease (MDRD) formula [11,12]:

$\mathrm{eGFR} = 186 \times \mathrm{Scr}^{-1.154} \times \text{age (years)}^{-0.203} \times 0.742 \text{ (if female)} \times 1.213 \text{ (if African American)}$

where Scr = serum creatinine (mg/dl); age in years; result in ml/min/1.73m²

Patients were classified as stage 3 (eGFR 30-59 ml/min/1.73m²), stage 4 (eGFR 15-29 ml/min/1.73m²), or stage 5 (eGFR < 15 ml/min/1.73m²). Patients with eGFR < 15 ml/min/1.73m² who were not yet initiated on dialysis were considered stage 5.

Thyroid function testing

All thyroid function tests were performed on fasting venous blood samples collected after a fast of 10-12 hours. Serum free triiodothyronine (FT3) and free thyroxine (FT4) were measured by competitive enzyme-linked immunosorbent assay (ELISA). Serum thyroid-stimulating hormone (TSH) was measured by sandwich ELISA. Reference ranges used were as follows: TSH: 0.35-5.1 μIU/ml; FT3: 1.80-4.20 pg/ml; FT4: 0.50-1.40 ng/dl. Thyroid status was classified as: (i) euthyroid: TSH, FT3, and FT4 all within reference ranges; (ii) OH: TSH > 5.1 μIU/ml with FT4 <0.50 ng/dl; (iii) SCH: TSH > 5.1 μIU/ml with normal FT4; (iv) low T3 syndrome: FT3 < 1.80 pg/ml with normal or low-normal TSH and FT4. All assays were performed in the central accredited laboratory of Dr. BSAH in accordance with the manufacturer's standard operating procedures.

Other laboratory investigations

Complete blood count, including Hb, was performed using an automated haematology analyser. Serum creatinine was measured by the modified Jaffé method. Blood urea was measured by the urease-diacetyl monoxime method. Normal reference ranges applied were as follows: Hb (males): 13.0-17.0 g/dl, (females): 12.0-16.0 g/dl; serum creatinine: 0.6-1.2 mg/dl; blood urea: 15-40 mg/dl. All laboratory investigations were performed in the central accredited laboratory of the institution.

Statistical analysis

Statistical analysis was performed using IBM SPSS Statistics version 20 (IBM Corp., Armonk, NY). Continuous variables are expressed as mean ± standard deviation (SD). Categorical variables are expressed as frequencies and percentages. One-way analysis of variance (ANOVA) was used to compare continuous variables across three or more groups; post-hoc pairwise comparisons were performed using the Bonferroni correction. An independent samples t-test was used for comparison between two groups. Chi-square (χ²) test and Fisher's exact test were used for categorical comparisons. Pearson's correlation coefficient (r) was calculated for bivariate associations between continuous variables. Binary odds ratios (OR) with 95% confidence intervals (CI) were calculated using Woolf's method for risk factor analysis. A two-tailed p-value ≤ 0.05 was considered statistically significant.

## Results

Baseline demographic and clinical characteristics

A total of 100 patients with confirmed CKD on conservative management were enrolled. The mean age of the cohort was 51.32 ± 11.89 years (range: 19-73 years), with the majority (33%) in the 50-60 year age group. There was a significant male preponderance, with 72% males and 28% females. Hypertension was the most common comorbidity (present in 52%: 32% HTN alone, 20% HTN + T2DM); T2DM alone was present in 14%, and 34% had no comorbidity. CKD stage 5 was the most prevalent stage (44%), followed by stage 4 (41%) and stage 3 (15%), reflecting the advanced disease burden in this hospital-based cohort (Table 1). Thyroid dysfunction was identified in 55% of patients. The most common abnormality was SCH (24%), followed by OH (22%), and low T3 syndrome (9%). Total hypothyroidism (overt + subclinical) was present in 46% of patients. No patient had biochemical evidence of hyperthyroidism.

**Table 1:** Baseline demographic and clinical characteristics of the study subjects (n = 100) SD: standard deviation; CKD: chronic kidney disease; eGFR: estimated glomerular filtration rate; HTN: hypertension; T2DM: type 2 diabetes mellitus; SCH: subclinical hypothyroidism; OH: overt hypothyroidism

Characteristic	Value
Age, years, mean ± SD	51.32 ± 11.89
Age group 50–60 years, n (%)	33 (33%)
Sex: male, n (%)	72 (72%)
Sex: female, n (%)	28 (28%)
CKD stage 3 (eGFR 30–59), n (%)	15 (15%)
CKD stage 4 (eGFR 15–29), n (%)	41 (41%)
CKD stage 5 (eGFR < 15), n (%)	44 (44%)
Hypertension only, n (%)	32 (32%)
HTN + T2DM, n (%)	20 (20%)
T2DM only, n (%)	14 (14%)
No comorbidity, n (%)	34 (34%)
Euthyroid, n (%)	45 (45%)
SCH, n (%)	24 (24%)
OH, n (%)	22 (22%)
Low T3 syndrome, n (%)	9 (9%)
Total hypothyroidism (OH + SCH), n (%)	46 (46%)

Gender-stratified analysis of thyroid dysfunction

The gender-specific analysis is presented in Table 2. Female patients had a markedly higher prevalence of hypothyroidism (71.4%, 20/28) compared to males (36.1%, 26/72; χ² = 10.335, p = 0.016). SCH was present in 39.3% of females versus 18.1% of males, and OH in 32.1% versus 18.1%, respectively. Euthyroidism was significantly more common in males (52.8% vs 25.0%). Female CKD patients had a significantly higher mean TSH (5.84 ± 2.95 vs 4.41 ± 3.04 μIU/ml; p = 0.036), while FT3, FT4, haemoglobin, serum creatinine, blood urea, and eGFR did not differ significantly between sexes (all p > 0.05), indicating that the greater thyroid burden in females was not explained by a greater degree of renal impairment. The OR for hypothyroidism in females versus males was 4.42 (95% CI: 1.71-11.44; p = 0.003), making female gender the most strongly associated variable related to hypothyroidism in this cohort.

**Table 2:** Gender-stratified analysis: thyroid status, laboratory parameters, and OR (n = 100) ^*^P < 0.05. ^**^P < 0.01 SCH: subclinical hypothyroidism; OH: overt hypothyroidism; SD: standard deviation; TSH: thyroid-stimulating hormone; FT3: free triiodothyronine; FT4: free thyroxine; Hb: haemoglobin; eGFR: estimated glomerular filtration rate; OR: odds ratio; CI: confidence interval (Woolf's method); χ²: chi-square; NS: not significant

Parameter	Male (n = 72)	Female (n = 28)	Test statistic	P-value
Euthyroid, n (%)	38 (52.8%)	7 (25.0%)	χ² = 10.335	0.016^*^
SCH, n (%)	13 (18.1%)	11 (39.3%)	—	—
OH, n (%)	13 (18.1%)	9 (32.1%)	—	—
Total hypothyroidism, n (%)	26 (36.1%)	20 (71.4%)	χ² = 10.335	0.003^**^
Low T3 syndrome, n (%)	8 (11.1%)	1 (3.6%)	—	—
Mean TSH, μIU/ml, mean ± SD	4.41 ± 3.04	5.84 ± 2.95	t = −2.116	0.036^*^
Mean FT3, pg/ml, mean ± SD	2.18 ± 0.71	2.09 ± 0.68	t = 0.556	0.578 (NS)
Mean FT4, ng/dl, mean ± SD	0.82 ± 0.25	0.78 ± 0.24	t = 0.693	0.490 (NS)
Mean Hb, g/dl, mean ± SD	9.22 ± 1.74	8.89 ± 1.79	t = 0.913	0.364 (NS)
Mean eGFR, ml/min/1.73 m², mean ± SD	18.14 ± 10.32	17.06 ± 9.84	t = 0.499	0.619 (NS)
OR for hypothyroidism (female vs male)	—	4.42 (95% CI: 1.71–11.44)	Woolf's method	0.003^**^

Renal and haematological profile across thyroid status groups

The complete laboratory profile stratified by thyroid status is presented in Table 3. Thyroid hormone values followed expected biochemical patterns: TSH was highest in OH (8.80 ± 2.41 μIU/ml), FT3 and FT4 were lowest in OH (1.26 ± 0.30 pg/ml and 0.40 ± 0.05 ng/dl, respectively; all ANOVA p < 0.001).

**Table 3:** Renal and thyroid laboratory profile by thyroid status ^*^P < 0.05. ^**^P < 0.01. ^†^Indicates the most clinically deviant group per parameter SD: standard deviation; SCH: subclinical hypothyroidism; OH: overt hypothyroidism; ANOVA: analysis of variance; TSH: thyroid-stimulating hormone; FT3: free triiodothyronine; FT4: free thyroxine; Hb: haemoglobin; eGFR: estimated glomerular filtration rate; NS: not significant

Parameter	Euthyroid (n =45), mean ± SD	SCH (n = 24), mean ± SD	OH (n = 22), mean ± SD	Low T3 (n = 9), mean ± SD	ANOVA F-value	ANOVA p-value
TSH, μIU/ml	2.68 ± 0.97	7.12 ± 1.84	8.80 ± 2.41^†^	2.34 ± 0.91	F = 112.4	< 0.001^**^
FT3, pg/ml	2.48 ± 0.42	2.19 ± 0.49	1.26 ± 0.30^†^	1.41 ± 0.28^†^	F = 47.2	< 0.001^**^
FT4, ng/dl	0.91 ± 0.18	0.84 ± 0.19	0.40 ± 0.05^†^	0.72 ± 0.14	F = 68.9	< 0.001^**^
Hb, g/dl	9.72 ± 1.37	9.47 ± 1.52	7.97 ± 1.86^†^	8.86 ± 1.44	F = 6.349	0.001^**^
Serum creatinine, mg/dl	4.20 ± 1.82	5.38 ± 2.94	7.01 ± 5.50	5.67 ± 3.21	F = 3.11	0.029^*^
Blood urea, mg/dl	98.79 ± 44.13	108.42 ± 51.20	152.03 ± 95.01^†^	136.67 ± 62.10^†^	F = 3.24	0.024^*^
eGFR (ml/min/1.73 m²)	19.84 ± 9.41	16.94 ± 9.02	13.42 ± 8.47	15.33 ± 7.94	F = 1.74	0.160 (NS)

Haemoglobin differed significantly across thyroid groups (ANOVA F = 6.349, p = 0.001). The OH group had the lowest mean Hb (7.97 ± 1.86 g/dl), significantly below the euthyroid group (9.72 ± 1.37 g/dl; post-hoc p < 0.001) and the SCH group (9.47 g/dl; post-hoc p = 0.003). A novel finding was the significantly elevated blood urea in the OH group (152.03 ± 95.01 mg/dl vs 98.79 ± 44.13 mg/dl in euthyroid patients; post-hoc p = 0.003; ANOVA p = 0.024), indicating that azotaemia is disproportionately severe in overtly hypothyroid patients with CKD. The low T3 syndrome group also demonstrated significantly higher blood urea than the euthyroid group (136.67 vs 98.79 mg/dl; post-hoc p = 0.036). Mean eGFR was lowest in the OH group (13.42 ml/min/1.73m²), but between-group differences for eGFR did not reach significance (p = 0.160) (Table 4).

**Table 4:** Bonferroni pairwise comparisons for Hb and blood urea by thyroid status ^*^P < 0.05. ^**^P < 0.01 Bonferroni correction applied. No separate test statistic is reported for post-hoc pairwise comparisons (derived from one-way ANOVA F-values in Table 3) Hb: haemoglobin; SCH: subclinical hypothyroidism; OH: overt hypothyroidism; T3: triiodothyronine; NS: not significant; ANOVA: analysis of variance

Comparison	Hb, g/dl, Bonferroni p-value	Blood urea, mg/dl, Bonferroni p-value
Euthyroid vs SCH	0.832 (ns)	0.832 (NS)
Euthyroid vs OH	< 0.001^**^	0.003^**^
Euthyroid vs low T3	0.195 (ns)	0.036^*^
SCH vs OH	0.003^**^	0.087 (NS)
SCH vs low T3	0.726 (ns)	0.284 (NS)
OH vs low T3	0.293 (ns)	0.846 (NS)

Laboratory profile across CKD stages

The laboratory profile by CKD stage is presented in Table 5. Haemoglobin declined progressively across stages (stage 3: 10.33 ± 1.69 g/dl; stage 4: 9.80 ± 1.51 g/dl; stage 5: 8.34 ± 1.43 g/dl; ANOVA F = 14.696, p < 0.001). Post-hoc analysis confirmed that stage 5 had significantly lower Hb than both stage 3 (Bonferroni p < 0.001) and stage 4 (Bonferroni p < 0.001), while stage 3 and stage 4 were not significantly different (Bonferroni p = 0.265). TSH showed statistically significant variation across stages (ANOVA F = 3.39, p = 0.037), with a non-linear pattern: stage 3 (5.24 μIU/ml), stage 4 (3.88 μIU/ml), stage 5 (5.53 μIU/ml). FT3 and FT4 did not differ significantly across stages. Serum creatinine, blood urea, and eGFR all demonstrated highly significant stage-dependent progression (all p < 0.001). Complete Bonferroni post-hoc comparisons for haemoglobin are presented in Table 6.

**Table 5:** Complete laboratory profile by CKD stage with ANOVA ^*^P < 0.05. ^**^P < 0.01. ^†^Indicates the most abnormal group per parameter CKD: chronic kidney disease; ANOVA: analysis of variance; SD: standard deviation; Hb: haemoglobin; TSH: thyroid-stimulating hormone; FT3: free triiodothyronine; FT4: free thyroxine; eGFR: estimated glomerular filtration rate; NS: not significant

Parameter	Stage 3 (n = 15), mean ± SD	Stage 4 (n = 41), mean ± SD	Stage 5 (n = 44), mean ± SD	ANOVA F-value	ANOVA p-value
Hb, g/dl	10.33 ± 1.69	9.80 ± 1.51	8.34 ± 1.43^†^	F = 14.696	< 0.001^**^
TSH, μIU/ml	5.24 ± 3.41	3.88 ± 2.74	5.53 ± 3.12	F = 3.39	0.037^*^
FT3, pg/ml	2.39 ± 0.54	2.22 ± 0.61	2.06 ± 0.58	F = 1.62	0.201 (NS)
FT4, ng/dl	0.88 ± 0.21	0.83 ± 0.24	0.80 ± 0.26	F = 0.31	0.731 (NS)
Creatinine, mg/dl	2.18 ± 0.52	4.40 ± 1.14	8.06 ± 4.22^†^	F = 59.8	< 0.001^**^
Blood urea, mg/dl	68.40 ± 22.14	103.68 ± 37.82	142.34 ± 72.18^†^	F = 22.4	< 0.001^**^
eGFR, ml/min/1.73 m²	44.28 ± 8.91	20.42 ± 4.17	8.84 ± 3.24^†^	F = 214.3	< 0.001^**^
Age, years	50.13 ± 12.40	51.88 ± 11.14	51.16 ± 12.32	F = 0.10	0.905 (NS)

**Table 6:** Bonferroni pairwise comparisons for HB by CKD stage ^**^P < 0.01 Bonferroni correction applied. Post-hoc comparisons are presented for Hb only (the sole parameter with a significant ANOVA result warranting pairwise testing for Hb). No separate test statistic reported for Bonferroni post-hoc comparisons HB: haemoglobin; CKD: chronic kidney disease; ANOVA: analysis of variance, NS: not significant

Comparison	Hb, g/dl, Bonferroni p-value
Stage 3 vs stage 4	0.265 (NS)
Stage 3 vs stage 5	< 0.001^**^
Stage 4 vs stage 5	< 0.001^**^

Correlation analysis and risk factor assessment

**Figure 1:**
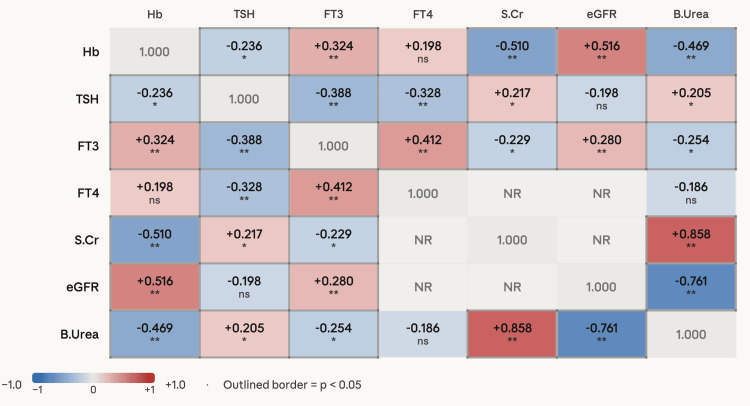
Pearson correlation matrix ^*^P < 0.05. ^**^P < 0.01 Diagonal: self-correlation (1.000). Cell shading: dark blue: strong negative (|r| ≥ 0.50); mid blue: moderate negative (|r| 0.30–0.49); light blue: weak negative (|r| < 0.30, sig.); corresponding reds for positive. Grey: not significant; pale grey: NR Hb: haemoglobin; TSH: thyroid-stimulating hormone; FT3: free triiodothyronine; FT4: free thyroxine; eGFR: estimated glomerular filtration rate; S.Cr: serum creatinine; B.Urea: blood urea; NR: pair not reported in original analysis

Pearson correlation coefficients and are presented in Figure 1 . Haemoglobin showed significant negative correlations with TSH (r = −0.236, p = 0.018), serum creatinine (r = −0.510, p < 0.001), and blood urea (r = −0.469, p < 0.001), and significant positive correlations with eGFR (r = 0.516, p < 0.001) and FT3 (r = 0.324, p = 0.001), establishing it as a marker that simultaneously reflects both renal and thyroid dysfunction severity. TSH was positively correlated with serum creatinine (r = 0.217, p = 0.030) and blood urea (r = 0.205, p = 0.040), and negatively correlated with FT3 (r = −0.388, p < 0.001) and FT4 (r = −0.328, p = 0.001). FT3 correlated negatively with creatinine (r = −0.229, p = 0.022) and blood urea (r = −0.254, p = 0.011). Comorbidities (T2DM, HTN) were not significantly associated with thyroid status (p = 0.16). Age was not significantly associated with thyroid status (ANOVA p = 0.18). Bivariate risk analysis identified two significant correlates of hypothyroidism (Table 7). Female gender conferred an OR of 4.42 (95% CI: 1.71-11.44; p = 0.003), and stage 5 CKD conferred an OR of 2.60 (95% CI: 1.15-5.85; p = 0.034) for hypothyroidism compared to stages 3+4.

**Table 7:** Risk factor analysis for hypothyroidism (n = 100) ^*^P < 0.05. ^**^P < 0.01 OR calculated by Woolf's method. Hypothyroidism: overt + subclinical hypothyroidism (n = 46 (46%)) OR: odds ratio; CI: confidence interval; CKD: chronic kidney disease; T2DM: type 2 diabetes mellitus; HTN: hypertension; NS: not significant

Predictor	OR	95% CI	Test statistic	P-value
Female gender	4.42	1.71–11.44	Woolf's method	0.003^**^
CKD stage 5 vs stages 3+4	2.60	1.15–5.85	Woolf's method	0.034^*^
T2DM (vs no T2DM)	1.42	0.62–3.24	Woolf's method	0.16 (NS)
HTN (vs no HTN)	1.18	0.54–2.59	Woolf's method	0.68 (NS)

## Discussion

Overall prevalence and pattern of thyroid dysfunction

The overall prevalence of hypothyroidism in our study was 46%, which is consistent with the higher estimates reported from hospital-based CKD cohorts in developing countries. Ramirez et al. first described low circulating T3 levels in uraemic patients as early as 1976 [7], and subsequent large-scale studies have documented a hypothyroidism prevalence ranging from 20% to 60% in CKD populations [4-6]. The dominance of subclinical hypothyroidism (24%) over overt hypothyroidism (22%) in our cohort reflects the gradual evolution of thyroid dysfunction as CKD advances, consistent with the findings of Lo et al., who demonstrated that TSH elevation precedes a clear reduction in FT4 levels in early to moderate CKD [13]. The low T3 syndrome, present in 9% of patients, represents a distinct non-thyroidal illness pattern, arising as a consequence of impaired peripheral deiodination of T4 to T3, as elaborated by Kaptein et al. [8], and reflects uraemia-induced suppression of type 1 deiodinase activity.

Gender as an independent predictor

The most striking finding of our study was the nearly twofold higher prevalence of hypothyroidism in female CKD patients (71.4%) compared to males (36.1%), with a fourfold odds ratio on bivariate analysis (OR: 4.42, p = 0.003). This finding is of particular clinical interest because female gender is already an established risk factor for autoimmune thyroid disease in the general population, with an estimated female-to-male incidence ratio of 5:1 to 10:1 [14]. However, the important distinction in our cohort is that patients with known hypothyroidism on treatment were explicitly excluded at the study design stage, thereby suggesting that the excess hypothyroidism detected in females likely represents newly identified or subclinical thyroid dysfunction associated with CKD-related mechanisms rather than pre-existing autoimmune disease. This interpretation is further supported by the significantly higher TSH in females (5.84 vs 4.41 μIU/ml, p = 0.036) despite comparable renal function parameters. Serum creatinine, blood urea, and eGFR were all similar between sexes, indicating that sex hormones or sex-specific differences in the regulation of the HPT axis may amplify the thyroid-suppressive effects of uraemia.

Carrero et al. demonstrated that female sex hormones modulate the clearance of TSH and alter the pituitary set point for TSH release, potentially rendering females more susceptible to TSH elevation under conditions of uraemic stress [15]. Similarly, Iglesias et al. noted that CKD progression is associated with disproportionate suppression of thyroid hormone levels in women, attributed in part to alterations in sex hormone-binding globulin and reduced androgen-mediated hepatic thyroid hormone production [16]. Our data add to this evidence by quantifying the risk differential as an OR of 4.42 in an Indian hospital-based cohort, a magnitude that supports consideration of thyroid function testing in female CKD patients, though prospective studies are needed to determine whether a formal screening strategy improves clinical outcomes.

Anaemia as a marker and consequence of thyroid dysfunction

The significant association between haemoglobin levels and thyroid status (ANOVA p = 0.001) in our study adds an important dimension to the understanding of multifactorial anaemia in CKD. Overt hypothyroidism patients had a mean Hb of 7.97 g/dl, substantially lower than the euthyroid group (9.72 g/dl, p < 0.001), and this difference was not explained by differences in CKD stage distribution alone. Multiple mechanisms may account for this relationship. Thyroid hormones directly stimulate erythropoiesis by upregulating erythropoietin gene expression and enhancing the sensitivity of erythroid progenitors to erythropoietin [17]. In the hypothyroid state, this stimulatory effect is lost, compounding the erythropoietin deficiency already present in CKD. Additionally, hypothyroidism reduces intestinal iron absorption and impairs vitamin B12 and folate metabolism [18], further deepening the anaemia.

The significant negative correlation between haemoglobin and TSH (r = −0.236, p = 0.018) and the positive correlation between haemoglobin and FT3 (r = 0.324, p = 0.001) observed in our data are consistent with these mechanistic pathways. Our finding that overt hypothyroidism was associated with the lowest haemoglobin values across thyroid status groups strongly suggests that thyroid dysfunction contributes to the anaemia burden of CKD, though the cross-sectional design does not permit a causal interpretation. This has practical implications. CKD patients with unexplained severe anaemia despite erythropoiesis-stimulating agent (ESA) therapy or iron supplementation may warrant evaluation for hypothyroidism as a potentially reversible contributory factor.

Azotaemia and its relationship with thyroid dysfunction

The significant elevation of blood urea in overt hypothyroidism (152.03 mg/dl vs 98.79 mg/dl in euthyroid patients, p = 0.003) represents, to our knowledge, one of the first formal quantifications of this association in a contemporary Indian CKD cohort. The positive correlation of blood urea with TSH (r = 0.205, p = 0.040) and its negative correlation with FT3 (r = −0.254, p = 0.011) suggest a biochemical link between azotaemia and thyroid dysfunction that goes beyond mere eGFR-mediated effects, since eGFR itself did not significantly differ across thyroid groups (p = 0.160).

The pathophysiological basis for this association is multifactorial. Hypothyroidism reduces renal plasma flow and GFR through decreased cardiac output and increased peripheral vascular resistance, leading to relative hypoperfusion of the nephron and reduced urea clearance [19]. Additionally, hypothyroidism reduces Na⁺-K⁺-ATPase activity, impairing tubular urea secretion [20]. The elevated urea in the low T3 syndrome group (136.67 mg/dl), in the absence of elevated TSH, further supports the view that peripheral thyroid hormone deficiency, independent of central HPT axis suppression, is associated with uraemia in CKD. Whether this relationship is bidirectional or primarily driven by renal dysfunction cannot be determined from this cross-sectional study.

Non-significant associations: comorbidities and age

Neither diabetes mellitus nor hypertension was independently associated with thyroid status (p = 0.16) or with TSH elevation in our cohort. While T2DM showed a borderline trend with TSH (p = 0.077), this did not reach statistical significance. These findings are consistent with those of Hardy and Raghunandam [21] and suggest that in the setting of CKD, the degree of renal impairment and gender are more strongly associated with thyroid dysfunction than the type of comorbidity. Age was also not significantly associated with thyroid status (p = 0.18), arguing against age-related subclinical hypothyroidism as a confounding factor in our findings.

Limitations

This study has several limitations. Its cross-sectional design precludes causal inference; while associations between gender, anaemia, azotaemia, and thyroid dysfunction are demonstrated, directionality cannot be established. No multivariate logistic regression was performed. With 46 outcome events among 100 participants, a five-predictor logistic regression model would not satisfy the minimum events per variable criterion (EPV ≥ 10), risking model overfitting and unreliable estimates. Bivariate odds ratios (calculated using Woolf’s method) are therefore reported and interpreted as measures of association, not independent effect. Thyroid peroxidase (TPO) antibody and thyroglobulin antibody status were not assessed, precluding the formal exclusion of subclinical autoimmune thyroid disease. The sample size of 100, while adequate for the primary aims, limits statistical power for subgroup analyses. Lastly, the study population was derived from a single tertiary care centre, which may limit its generalisability to primary care CKD populations.

## Conclusions

Thyroid dysfunction is highly prevalent in CKD patients on conservative management, with hypothyroidism, including both overt and subclinical types, affecting nearly half of this cohort. Female gender and advanced CKD stage 5 show the strongest associations with hypothyroidism on bivariate analysis, and OH is associated with more severe anaemia and higher azotaemia beyond what is explained by CKD stage alone. These findings support the hypothesis that targeted thyroid function testing in CKD patients may identify previously undetected thyroid dysfunction; prospective studies are warranted to evaluate the clinical benefit and cost-effectiveness of such a strategy.
